# Enhanced Radiation Tolerance of Tungsten Nanoparticles to He Ion Irradiation

**DOI:** 10.3390/nano8121052

**Published:** 2018-12-14

**Authors:** Emily Aradi, Jacob Lewis-Fell, Robert W. Harrison, Graeme Greaves, Anamul H. Mir, Stephen E. Donnelly, Jonathan A. Hinks

**Affiliations:** 1 School of Computing and Engineering, University of Huddersfield, Queensgate, Huddersfield HD1 3DH, UK; Jacob.Lewis-Fell2@hud.ac.uk (J.L.-F.); r.w.harrison@manchester.ac.uk (R.W.H.); G.Greaves@hud.ac.uk (G.G.); A.H.Mir@hud.ac.uk (A.H.M.); s.e.donnelly@hud.ac.uk (S.E.D.); j.a.hinks@hud.ac.uk (J.A.H.); 2 School of Mechanical, Aerospace and Civil Engineering, University of Manchester, Sackville Street, Manchester M1 3NJ, UK

**Keywords:** plasma-facing materials, nanoporous materials, tungsten nanoparticles, radiation tolerance, in-situ TEM, helium bubbles

## Abstract

Materials exposed to plasmas in magnetic confinement nuclear reactors will accumulate radiation-induced defects and energetically implanted gas atoms (from the plasma and transmutations), of which insoluble helium (He) is likely to be the most problematic. The large surface-area-to-volume ratio exhibited by nanoporous materials provides an unsaturable sink with the potential to continuously remove both point defects and He. This property enhances the possibilities for these materials to be tailored for high radiation-damage resistance. In order to explore the potential effect of this on the individual ligaments of nanoporous materials, we present results on the response of tungsten (W) nanoparticles (NPs) to 15 keV He ion irradiation. Tungsten foils and various sizes of NPs were ion irradiated concurrently and imaged in-situ via transmission electron microscopy at 750 °C. Helium bubbles were not observed in NPs with diameters less than 20 nm but did form in larger NPs and the foils. No dislocation loops or black spot damage were observed in any NPs up to 100 nm in diameter but were found to accumulate in the W foils. These results indicate that a nanoporous material, particularly one made up of ligaments with characteristic dimensions of 30 nm or less, is likely to exhibit significant resistance to He accumulation and structural damage and, therefore, be highly tolerant to radiation.

## 1. Introduction

The prospect of using nuclear fusion for energy production has motivated a large research effort with the aim of finding suitable materials to be used for fusion reactors [1]. ITER, currently under construction, is a thermonuclear project that will demonstrate the use of magnetic confinement to sustain a plasma of sufficiently high temperature to induce the fusion of deuterium and tritium for significantly longer periods and on a larger scale than in previous reactors such as the Joint European Torus (JET) and Toroidal Fusion Test Reactors (TFTR) [1,2]. Although such reactors will be designed to contain the plasma as much as possible, the surrounding materials are still expected to encounter extreme conditions of high irradiation fluxes and large heat loads in the range of 8–10 MW·m^−2^ [3,4]. Tungsten (W) has been proposed as one of the most suitable candidates to be used for divertor armour (a plasma-facing component) in ITER and for the first-wall in the DEMOnstration (DEMO) power station projects [5,6,7,8]. This has been inspired by its superior characteristics including high melting temperature (3700 K), good mechanical properties (yield strength of order 750 MPa), outstanding thermal conductivity (170 W·m^−1^·K^−1^ at room temperature), and low sputter yield–all essential for the anticipated plasma environments in these reactors [9].

During ion irradiation as a means to simulate reactor conditions, the incident particles interact with the atoms of the target material creating displaced atoms which can aggregate in the form of point defect clusters that can grow to form extended defects such as dislocation loops [10,11]. As a result of neutron-induced (n,α) reactions, helium (He) is generated and is insoluble in most solids including W [12]. In the absence of other sinks, these He atoms will trap in regions of low electron density such as vacancies which can act as nucleation sites for the formation of He gas nanobubbles [13]. The accumulation of such nanobubbles may result in blistering, embrittlement, and dimensional alterations such as swelling—potentially leading to the failure of the material [14,15,16]. Where a material has a large concentration of sinks that can remove point defects and He, the nucleation and growth of extended defects such as dislocation loops and He bubbles is likely to be suppressed giving the material the potential for increased radiation resistance [10,11,16,17,18]. Some of the approaches that have been used to engineer radiation-resistant materials include the incorporation of high densities of sinks such as grain boundaries in nanocrystalline materials or nanostructures in nanolayered composites and oxide-dispersion-strengthened (ODS) steels [17,19,20]. In their studies, El-Altwani et al. [21,22,23] reported an improved radiation tolerance in nanocrystalline W compared with ultrafine-grained W, indicating an important effect of grain size (and thus grain boundary density) on radiation resistance. Ukai et al. [20] observed ductility retention and reduced swelling after He ion irradiation by adding nanocomposites to ferritic steel. Similarly, Misra et al. [17] induced ultra-high strengths and enhanced radiation tolerance by decreasing the diffusion length to the nearest sink via tailoring the layer thickness in nanolayered Cu-Nb composites.

Despite improved radiation resistance, nanocrystalline and nanolayered materials tend to lose their nanoscale structural features due to ion-induced mixing [24] and high-temperature-induced grain growth (above around 1300 °C in W), [25,26], posing a challenge for their use for extended periods in such extreme environments. Furthermore, the accumulation of He bubbles at grain boundaries may also increase the embrittlement of nanocrystalline materials in reactors [19,23]. As in a nanoporous material, both He and vacancies have a higher probability of reaching a surface before they can nucleate into nanobubbles (and similarly for point defects and the formation of structural defects) there is a potential for enhanced radiation resistance which needs to be explored [27]. For example, Li et al. [28], in their study of nanopore shrinkage in gold (Au), observed a significantly-lower dislocation loop density in nanoporous Au compared to coarse-grained Au under 1 MeV Kr irradiation at room temperature, indicating improved radiation tolerance.

The structure of a nanoporous material is an interconnected network of ligaments which can be considered individually as nanoparticles [29]. As nanoporous W materials are not currently commercially available, W NPs have been used as a model system in the work reported here. These NPs present the added advantage of allowing the isolation of a single “ligament” for in-situ transmission electron microscopy (TEM) observation during ion irradiation experiments without the influence of other surrounding structures (which could, for example, cause the deposition of sputtered material onto the region of interest or shadowing of the ion and/or electron beams).

Knowledge of the behaviour of nanoporous materials in a nuclear environment is still limited, with only a few theoretical studies carried out looking into the irradiation of NPs [11,30,31,32]. A recent review noted the lack of experimental studies regarding the size effect on radiation damage in nanoporous metals [10]. In the current work, we present novel results using in-situ TEM to compare the response of individual W nanoparticles (NPs) of different diameters and W foils to displacing He irradiation at the fusion-reactor-relevant temperature of 750 °C. We report on the bubble populations considered in terms of the size distributions and volumetric number densities (referred to simply as “bubble density” for brevity hereafter) as well as the sizes and volumetric number densities of dislocation loops created during the ion irradiation.

## 2. Methodology

### 2.1. Experimental

Tungsten NPs with 99.9% purity were obtained from American Elements (Los Angeles, CA, USA, product code: W-M-03M-NP.100P) [33] with diameters ranging typically from 20–100 nm. In order to allow side-by-side studies of the radiation effects with the tungsten foils, the NPs were randomly dispersed onto electrochemically polished W by dipping the foil in W nanopowder. The W foil performed the dual purpose of a comparative material as well as acting as a support providing good thermal contact to the NPs, especially, compared to alternatives such as carbon films which are prone to deterioration under the ion/electron beams and can serve as a possible source of contamination.

For the foil samples, W was obtained from Alfa Aesar (Haverhill, MA, USA, product code: CAS-7440-33-2) in the form of 0.1 mm sheets. Discs of 3 mm diameter were cut using a Gatan Model 3195 disc punch, then annealed to 1400 °C for 5 h in a vacuum (10^−1^ Pa) to remove any pre-existing effects from cold working. Final thinning was performed by electropolishing the samples with 0.5 wt.% NaOH aqueous solution using a Tenupol-5 obtaining electron-transparent regions of about 50 nm thickness. The thickness of the foils was measured using electron energy-loss spectroscopy (EELS) and thickness mapping performed using energy-filtered TEM (EFTEM) [34] taking the electron mean-free-path, *λ*, for W at room temperature as 15.5 nm [35]. After dispersion of the NPs onto the W disc, the samples were annealed in a TEM to 1000 °C using a Gatan Model 652 double-tilt heating holder to ensure the stability of the NPs at the edge of the foil at high temperature. Note that only NPs that protruded from the edge of the thinned region of the W foil (and, similarly, regions of the W foil clear of NPs for comparison) were selected for analysis to avoid potential ion and/or electron beam shadowing effects.

All the irradiations and observations of the evolution of the microstructure were performed at the Microscopes and Ion Accelerators for Materials Investigations (MIAMI) facilities using the MIAMI-2 system located at the University of Huddersfield. MIAMI-2 consists of a 350 kV National Electrostatics Corporation (NEC) ion accelerator (Middleton, WI, USA) coupled with a Hitachi H-9500 TEM (Tokyo, Japan) in which the ion beam is incident on the sample at 18.7° to the electron beam. The microscope was operated at 300 kV and the electron beam was turned off during irradiation steps in order to avoid synergistic effects between the electron and ion beams. The W samples were irradiated with 15 keV He^+^ ions at a flux of 10^13^ ions·cm^−2^·s^−1^ to a maximum end fluence of 1.1 × 10^17^ ions·cm^−2^. Experiments were performed at 750 °C, which is within the anticipated in-service temperature range for the plasma-facing divertor of ITER [8].

The Stopping and Range of Ions in Matter (SRIM) Monte Carlo computer code [36] was used to calculate the damage density and ion distribution for 15 keV He ions in W. A material in SRIM is calculated to have an infinite length in the Y and Z direction. The X direction is the user-defined thickness of the material. This approach works well when calculating for thin film materials, where the Y and Z dimensions are very large in comparison to the thickness X. However, when applying this approach to nanoparticles collision events, they may be calculated and recorded in volumes that a NP would not occupy, therefore, creating erroneous results. To correct for this, a Python script named Spherical Ion Calculation Modifier (SICMod) has been developed. This script scans the entry point of the ions across one hemispherical surface of a sphere of user-defined diameter. It then ignores all damage events that occur outside of that sphere; i.e., wherever a collision cascade leaves the sphere, all collisions that would subsequently occur along the exiting branch of the cascade in a conventional SRIM calculation are discarded. Finally, the script outputs colour maps showing the damage and implantation densities across the sphere. The SRIM calculations were performed following the standard procedure suggested by Stoller et al. [37] using the ‘Quick’ Kinchin–Pease mode for 1000 He ions with a target density of 19.3 g·cm^−3^. A displacement energy of 90 eV [38] was used with the lattice and surface binding energies both set to 0 eV [37].

**Figure 1:**
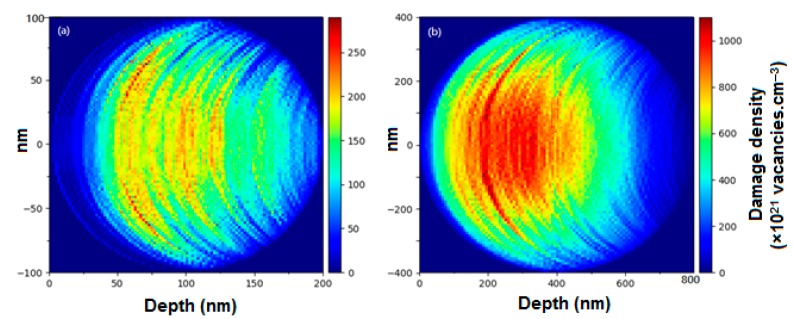
SRIM calculations modified using the SICMod code for a circular cross-section of spherical W NPs irradiated with 15 keV He to 1.1 × 10^17^ ions·cm^−2^ showing the damage density for diameters of (**a**) 20 nm and (**b**) 80 nm. (The colour scale units of damage density apply to both images.)

Figure 1 shows colour maps illustrating the damage density distributions in NPs with diameters of 20 and 80 nm irradiated with 15 keV He to a fluence of 1.1 × 10^16^ ions cm^−2^, indicating a lower damage density in the smaller NPs under these conditions.

### 2.2. Analysis

Nanoparticles of various diameters (binned with a class interval of 10 nm) were analysed and their volumes calculated assuming a spherical shape (confirmed by tilting in the TEM). The number of observable bubbles was divided by this volume to give the bubble density for each NP size. A similar procedure was followed for the purposes of comparison with the bulk material: A circular area (as seen in projection in the TEM) was selected in a region with the appropriate thickness to give a cylindrical volume equivalent to the volume of a given NP and, again, the number of observable bubbles was used to calculate the bubble density. NP and bubble diameters were determined using the ImageJ (FIJI) image analysis software [39]. In the case of NP diameters, these were calculated from measurements of the projected area at three different x-tilts (0° and ±30°) in the TEM.

## 3. Results and Discussion

### 3.1. Bubble Density

**Figure 2:**
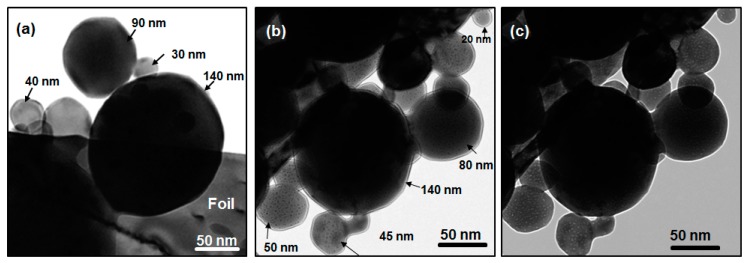
Bright-field TEM images showing the distribution of bubbles in W NPs of different sizes after irradiation with 15 keV He to a fluence of 9.6 × 10^16^ ions·cm^−2^ at 750 °C showing: (**a**) a distribution of NPs of different sizes dispersed at the edge of the electropolished region of a W foil before irradiation; (**b**) 800 nm overfocus; and (**c**) 800 nm underfocus after irradiation. Small bubbles appear as black spots in overfocus and white spots in underfocus.

Figure 2a is a bright-field TEM (BF-TEM) image illustrating a distribution of NPs of different sizes dispersed at the edge of an electropolished region of a W foil sample before irradiation. Figure 2b,c show overfocused and underfocused BF-TEM images, respectively, of W NPs of different sizes after irradiation to a fluence of 9.6 × 10^16^ ions·cm^−2^, demonstrating a distribution of He bubbles which appear as dark spots in overfocus and bright in underfocus due to Fresnel contrast. The figures give a general overview of the distribution and densities of bubbles found in NPs of different sizes in this work. The NPs remained morphologically stable under irradiation, indicating that any sputtering was below the level detectable in these experiments.

**Figure 3:**
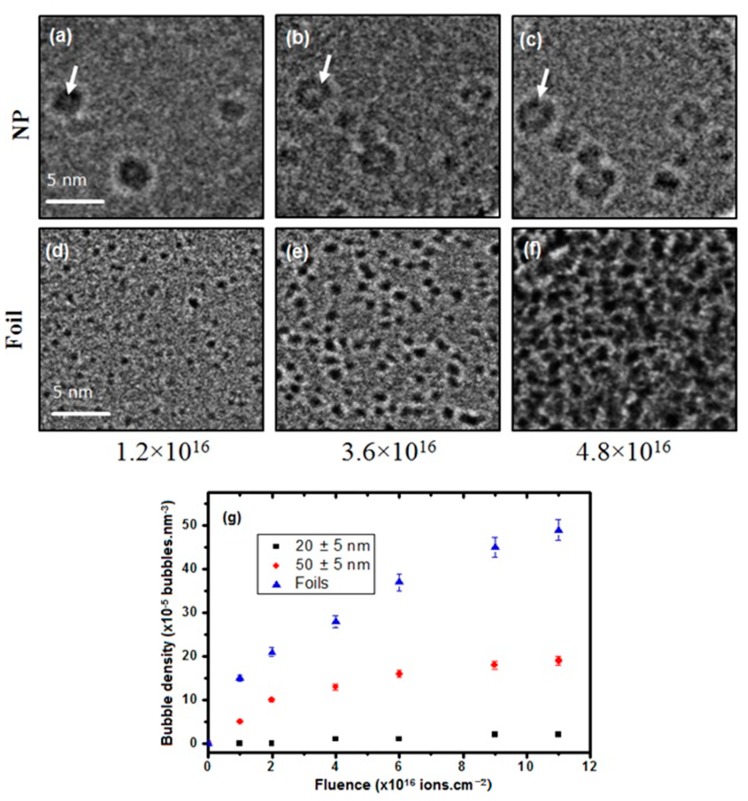
BF-TEM images comparing bubbles in a 35 nm diameter W NP (**a**–**c**) and foil (**d**–**f**) as a function of fluence taken at 400 nm overfocus. The arrows in (**a**–**c**) highlight the growth of a bubble in the NP as the fluence increases. (Scale marker in (**a**,**d**) applies to all images). Figure (**g**) shows the relationship between bubble density and fluence for NPs with diameters of 20 ± 5 and 50 ± 5 nm in the foil.

Figure 3a–e are high-magnification images comparing the bubble distribution in a 35 nm diameter NP and the foil as a function of fluence. Figure 3a,d presents images of samples irradiated to 1.2 × 10^16^ ions·cm^−2^, Figure 3b,e of samples irradiated to 3.6 × 10^16^ ions·cm^−2^ and Figure 3c,f of samples irradiated to 4.8 × 10^16^ ions·cm^−2^. The images show an increase in the bubble density for both the NP and the foil as a function of fluence with a larger concentration of bubbles in the foil compared to the NP. With increasing fluence, there is also a uniform distribution of the bubbles in the foil as opposed to the uneven distributions between the NPs with some having no bubbles. Figure 3g gives a comparison of the average bubble density in 20 and 50 nm diameter NPs and the foil as a function of fluence. A total of 22 NPs and 22 different regions in the foils were analysed for each size class interval. Under the irradiation conditions used here, 20 nm diameter NPs showed a very low degree of bubble accumulation, as shown in Figure 3g, with an average of <5 × 10^−5^ bubble·nm^−3^ at 1.1 × 10^17^ ions·cm^−2^. The evolution of the bubble density in the larger diameter NPs was observed to occur in three distinct regimes: First, an increase in bubble density with fluence; then, a saturation of 1.5 × 10^−5^ bubbles·nm^−3^ at 4.0 × 10^16^ ions·cm^−2^; and, finally, no further increase in the bubble density was observed up to the end fluence of 1.1 × 10^17^ ions·cm^−2^. For the foil, there was a linear increase in the average bubble density with increasing fluence up to 1.1 × 10^17^ ions·cm^−2^.

During irradiation, vacancy and interstitials will both have been formed as the W atoms were displaced from their lattice sites by the He ions. Interstitials are highly mobile compared to vacancies in W at 750 °C [22] and can rapidly diffuse to nearby sinks leaving an excess of vacancies in the matrix. Vacancies can act as nucleation sites trapping migrating He atoms to form He-vacancy (He-V) complexes [11,40] which can continue to grow with increasing fluence and eventually become nanobubbles visible in the TEM. The removal of He and vacancies as they escape via the free surface in a NP will result in fewer nucleation sites for He-V complexes and manifests itself as lower bubble densities in the NPs compared to the foil. The bubble density saturation at a fluence of 4 × 10^16^ ions·cm^−2^ in the 50 nm diameter NPs implies that this is a critical fluence above which all He and vacancies are absorbed by existing bubbles or escape via the surface under these conditions. For the foil samples, because of the greater distance to the nearest sink, there will be a lower probability of He and vacancies diffusing to the surface. This relatively greater degree of vacancy accumulation, in turn, creates more sites for He bubble nucleation in the foil, leading to the higher bubble density observed in the W foil in Figure 3.

Theoretical studies on nanomaterials by Bai et al. [41] indicate an increased defect accumulation in the matrix with decreased boundary density. Rajan et al. [42] also suggested that there is a depletion of vacancies due to grain boundaries that act as defect sinks resulting in a decreased bubble density in nanostructured austenitic stainless steel under He ion irradiation. The shorter distance to the surface in a NP facilitates the annihilation of vacancies via the ingress of interstitials without the need for the vacancy to migrate all the way to the sink as suggested by the modelling work of [31]. Bringa et al. [18], in their molecular dynamics (MD) simulations on the irradiation of nanoforms, concluded that defect migration to a ligament surface occurs faster than the time between cascades at typical fluxes, resulting in reduced damage accumulation. Using TEM, the same study reported a reduced bubble density in nanoporous Au irradiated with 45 keV Ne compared to the bulk with ligaments below 35 nm in diameter displaying no damage accumulation. Similarly, it has been reported that He irradiation of Fe nanocrystals resulted in bubble accumulation dependent on crystal size with smaller crystals accumulating He bubbles at lower concentrations and with reduced irradiation hardening compared to the bulk [43]. El-Atwani et al. [21,44] observed reduced bubble density in nanocrystalline W grains (<100 nm grain sizes) compared to ultrafine W (100–500 nm grain size range) which they attributed to the proximity of the grain boundary in nanocrystalline W acting as a sink for defects. These studies indicate that the effectiveness of interphases will depend on the distances defects migrate within the boundary planes and thus a shorter required distance in smaller crystals enhances the recombination of point defects at sinks.

**Figure 4:**
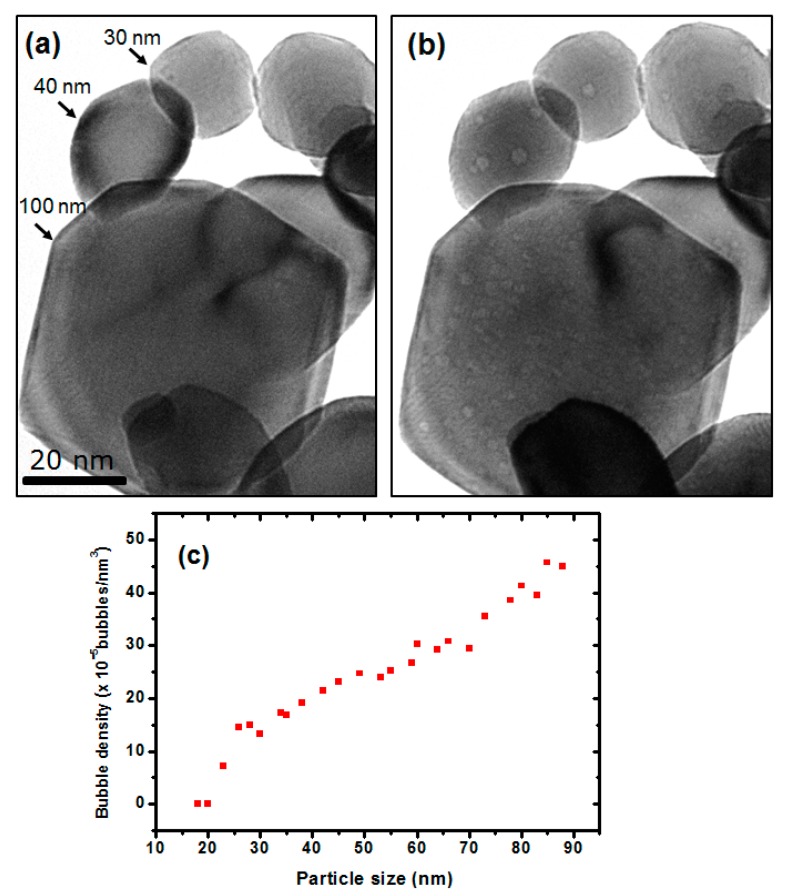
Underfocus BF-TEM images for W NPs: (**a**) unirradiated and (**b**) irradiated to a fluence of 1.1 × 10^17^ ions·cm^−2^ showing the bubble distributions in NPs of different sizes. The relationship between bubble density and particle size for W NPs with different diameters irradiated to a fluence of 1.1 × 10^17^ ions·cm^−2^ is shown in (**c**). (The scale marker in (**a**) applies to both images.)

To investigate the effect of NP size on sink efficiency, bubble densities in NPs of different sizes were analysed. Figure 4a shows a 400 nm underfocus BF-TEM image illustrating variations in bubble density for different NP sizes. It was observed that the 100 nm NP shown has a higher bubble density than the NPs with smaller diameters. The smallest NP size indicated in Figure 4b is approximately 30 nm in diameter and it has only one observable bubble. Figure 4c shows the relationship between bubble density and NP size with the largest NPs demonstrating a significantly higher bubble density than the smallest NPs.

The smaller the size of a NP the larger the surface-area-to-volume ratio. This decreases the probability of He-V complex formation due to the depletion of vacancies (as discussed above) and leads to a reduced rate of He retention as most of the He escapes via the large surface, resulting in a low bubble density in small NPs. As the size of the NP increases, the average distance to the surface lengthens; thus increasing the opportunities for bubble nucleation resulting in higher bubble densities. When the NPs were sufficiently large (typically >100 nm) it was observed that they behaved as the foil in these regards. Ultimately, fewer bubbles in the NPs means fewer obstacles to dislocation motion meaning the embrittlement of the NPs is likely much lower than that in the foil.

### 3.2. Bubble Size and Swelling Due to Bubbles

**Figure 5:**
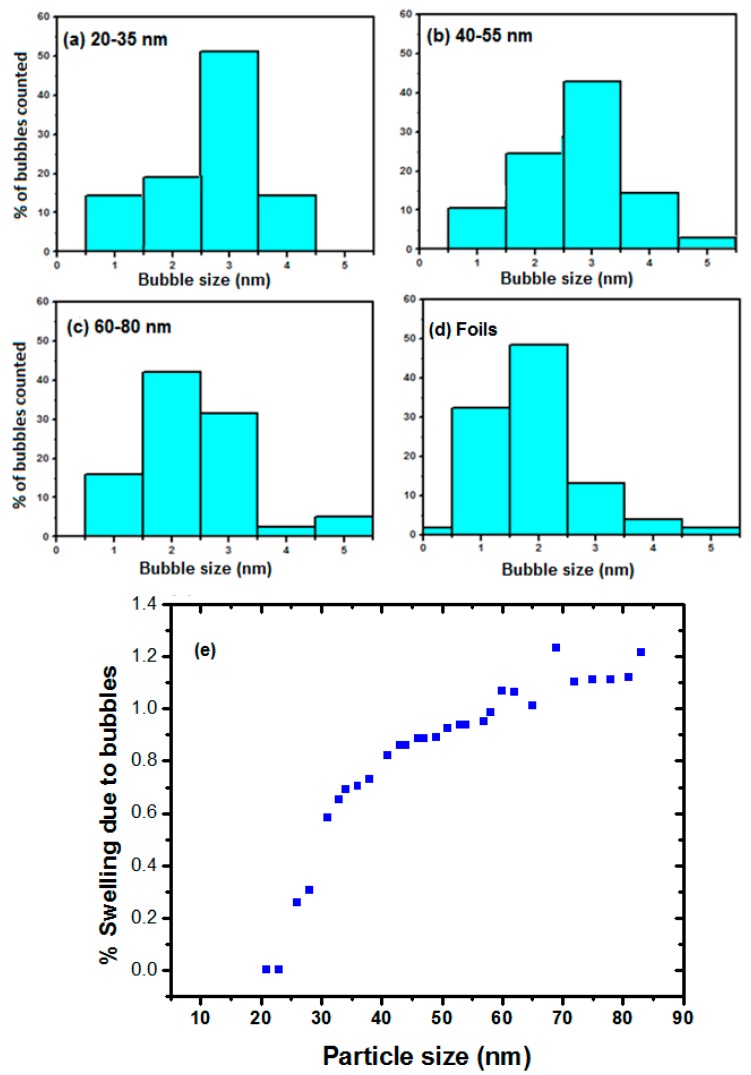
Distribution of bubble sizes in W irradiated with 15 keV He to a fluence of 1.1 × 10^17^ ions·cm^−2^: (**a**) 20–35 nm NPs (sample = 80 bubbles in 20 NPs); (**b**) 40–55 nm (sample = 200 bubbles in 20 NPs); (**c**) 60–80 nm (sample = 280 bubbles in 28 NPs); and (**d**) the foils (sample = 280 bubbles). (**e**) Plot of the size dependence of swelling due to bubbles for W NPs of different diameters.

As well as the bubble density, bubble size was also investigated as a function of NP diameter. The white arrows in Figure 3 indicate one example of significant bubble growth in a NP relative to that observed in the foil. This was a consistent trend, with fewer larger bubbles in the NPs compared to the bubbles in the foil specimens. Figure 5 shows the bubble size statistical distributions in various sizes of NP and in the foils with the largest bubbles observed in the smallest NPs. A total of 20 NPs and 20 different regions in the foil were analysed for each class interval. For distribution analysis, 80 bubbles in the 20–35 nm NP size range, 200 bubbles in the 40–55 nm range and 280 bubbles in the 60–80 nm range were analysed. The smaller number of bubbles counted in the 20–35 nm interval was due to the low bubble density and relatively low number of NPs per sample in this category. Note that no bubbles were observed for NPs with diameters below 20 nm under the experimental conditions reported here.

With decreasing NP size, the effect of an increasingly large surface-area-to-volume ratio and reduced rate of retention in the NPs appears to cause a lower density of larger bubbles. Two mechanisms may explain this behaviour. Firstly, as discussed above, the depletion of vacancies due to annihilation at the surface reduces the chance of new He-V complexes forming in a NP. It is reasonable to assume that as the He concentration increases, there is a high probability that the already-nucleated bubbles will capture a migrating He atom (He_x+1_–V_y_) and, as a result, the bubbles in small NPs will become larger. With increasing NP size, the distances point defects must migrate to reach the surface increases and hence defect annihilation at the surface reduces; this leads to an increase in the vacancy population in the matrix which can act as new nucleation sites to trap He and thus results in a larger number of smaller bubbles. Secondly, at 750 °C He–V clusters are highly mobile in W with a migration energy of 3 eV [21]. Therefore, there is an increasing probability of agglomeration of these complexes with decreasing NP size due to the shorter distances to the surface, resulting in larger bubbles compared to those in the larger NPs and the foil.

The effects on the NPs due to the formation of bubbles can be quantified by calculating the swelling due to their inclusion, assuming that the atoms they have displaced that have become interstitials or surface atoms have not left the NP and that each adds one atomic volume to the NP. The amount of NP swelling was calculated by evaluating the change in volume, Δ*V*, represented by the bubbles, *N*_B_, of radius, *r*, which were observable in the TEM and normalising to the volume of the NP, *V*, thus [45,46]:

(1) $$\frac{\Delta V}{V}=\frac{4\pi }{3V}\overset{N_{B}}{\underset{i}{\sum }}r_{i}^{3}$$

Figure 5d presents the percentage swelling of the NPs as a function of NP size. The swelling behaviour indicates that NPs with larger volumes tended to have higher relative swelling compared to smaller NPs; those with an average diameter of 20 nm had the smallest swelling of ˂0.5% and hence, the highest swelling resistance. For comparison, the actual volume change was determined by measuring the volume of the NPs before and after irradiation. Using 11 NPs with diameters between 70–100 nm, the average volume change was measured to be 1.3%, which agrees well with the calculated 1.2% volume change (swelling) due to bubbles. Since the change was within error margin in NPs with diameters <60 nm, the experimental measurements were not included.

### 3.3. Helium Concentration in NPs

In the absence of other sinks, it is assumed that most of the implanted He gas goes to vacancy sites to form He-V complexes and He bubbles. With the proximity to the surface in the NPs, a larger proportion of the He can escape to the surface. The pressure in the bubbles can be estimated (assuming the bubbles are at equilibrium since the experiments were done at high temperature) and hence used to determine the He contained in the bubbles for different NP sizes [47]. The internal gas pressure, *p*, required to balance the surface tension, *γ* (=2.5 Nm^−1^ for W [47]), of a spherical bubble of radius, *r*, at equilibrium in a solid is given by:

(2) $$p=\frac{2\gamma }{r}$$

**Figure 6:**
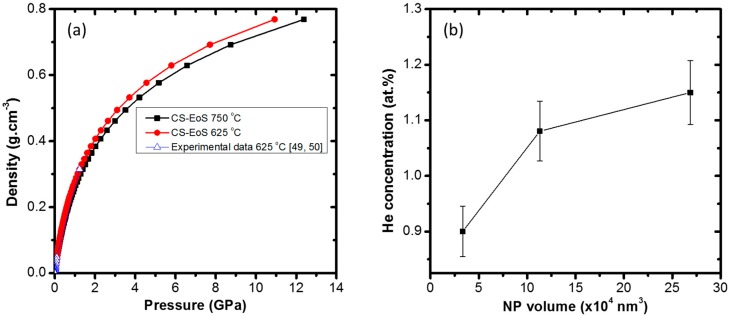
Plots of: (**a**) calculations and experimental data [49,50] for density as a function of pressure for He at 625 °C and 750 °C; and (**b**) relationship between He concentration (based on resolvable bubbles) and NP volume at a fluence of 1.1 × 10^17^ ions·cm^−2^.

Using the average bubble diameters, the pressure in the bubbles was calculated using Equation (2). For the relationship between the pressure and density of He, the Carnahan–Starling equation of state (CS-EoS) [48] in conjunction with available experimental data (for validation) were used. The results of the CS-EoS at 625 °C (for which some high-pressure experimental data [49,50] are available for comparison) and at 750 °C (which corresponds to the experiments reported here) are shown in Figure 6a.

Since there is good agreement between the predictions of CS-EoS and the experimental data at 625 °C, it can be assumed that the predictions of CS-EoS at 750 °C are also reasonable (the densities at 750 °C are about only 7% lower than those at 625 °C) as shown in Figure 6a. Using the CS-EoS at 750 °C, the densities of He in the bubbles corresponding to the pressures given by Equation (2) for different bubble sizes were calculated and are shown as the green plot in Figure 6a. The molar volume of the He gas in the bubbles was then estimated to be 8.2 cm^3^·mol^−1^ for the 40 nm diameter NPs with a 3.0 nm average bubble size and 7.3 cm^3^·mol^−1^ for the 80 nm diameter NPs with an average bubble diameter of 2.4 nm (corresponding to He/V ratios of 1.3 and 1.15, respectively). This agrees well with the He/V ratio in [47] for W and also with the deduction that there will be more He/V in NPs of smaller diameter than in larger NPs as discussed above.

Using the molar volume and bubble density, the He concentration in the bubbles was estimated and is plotted as a function of NP volume in Figure 6b. The concentration was estimated to be ~0.9 at.% in the 40 nm diameter NPs with the concentration increasing with NP size, implying less accumulation in smaller NPs. From SRIM calculations, an average He concentration of 2.2 at.% was estimated for a 80 nm diameter NP, which deviated from the ~1.1 at.% experimental value derived by the total volume of observable bubbles. This inconsistency of 1.1 at.% is most likely attributable to the He in He-V complexes and small bubbles which are not resolvable in the TEM and the He which has escaped via the surface.

### 3.4. Dislocation Loops

**Figure 7:**
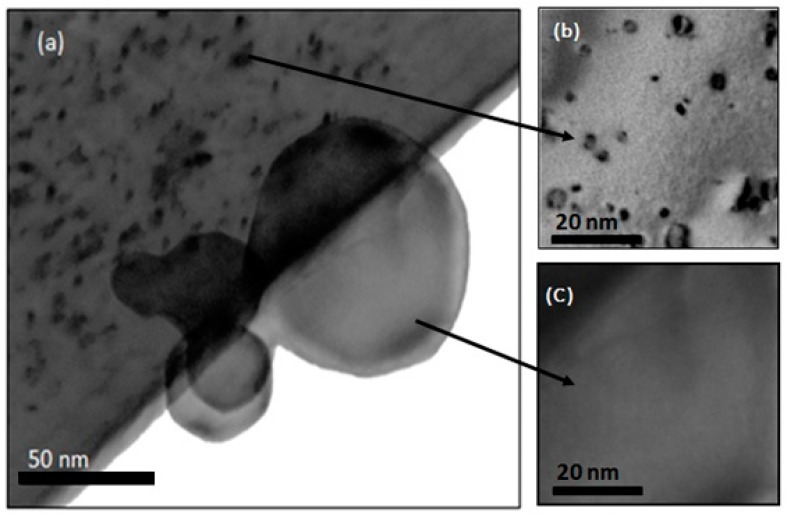
TEM images of: (**a**) NPs of different sizes and W foil irradiated to 1.1 × 10^17^ ions·cm^−2^; (**b**) enlarged area of the foil after tilting >15° to be close to a different zone axis to that shown in (**a**) to avoid any possible ***g***.***b*** invisibility criteria; and (**c**) enlarged area of NP after equivalent tilting of >15°. Note the complete absence of dislocation loops in the NPs evident in (**a**,**c**).

Figure 7a is a BF-TEM image of a foil and NPs irradiated to a fluence of 1.1 × 10^17^ ions·cm^−2^. Figure 7b,c are at-focus images of selected areas of foil and NP, respectively, illustrating clear differences between the damaged microstructures. Defect clusters and dislocation loops were observed in the foil with the sizes varying from 2–15 nm and an average size of 6.8 nm for 84 loops counted in different regions within the foil. In their study of the effects of He accumulation and atomic displacements on the microstructure of polycrystalline W, Harrison et al. [51,52] observed and characterised dislocation loops similar to those in the current work which formed under similar irradiation conditions. They determined the loops to be predominantly interstitial type with a Burgers vector of ***b*** = ½<111>.

For all the experimental conditions used in the current work, no NPs of any size studied exhibited defect clusters or dislocation loops, suggesting that their large surface-to-volume ratio increased defect annihilation leading to improved radiation resistance compared to the foil. (The NPs were tilted in the TEM by at least 15° to another zone axis to ensure that loops were not hidden by the ***g***.***b*** invisibility criterion).

## 4. Conclusions

Information on the effect of radiation damage on nanoporous materials and especially NPs is still limited. In the novel study reported here, in-situ TEM experiments have shown that, compared to W foil specimens, the free surfaces of W NPs act as effective point defect sinks successfully minimising He accumulation and stopping the formation of extended defect clusters such as dislocation loops. The NP sizes played an important role in determining the accumulation of He bubbles with NPs that had diameters <20 nm featuring no bubbles. In reactor environments, damage introduced in the form of dislocation loops, point defects and He from transmutation reactions results in embrittlement and dimensional changes that degrade the mechanical and structural properties of materials. Therefore, the ability to remove radiation-induced point defects and control He bubble nucleation and growth are crucial to improving the mechanical performance of irradiated materials. The lower bubble density and lack of dislocation loops observed in this study of W NPs are of significance for delaying the degradation of mechanical properties of W under such conditions. By engineering the size and geometry of the ligaments of nanoporous W, it should be possible to control the microstructural response by utilising the large surface as an effective sink for He release and point defect annihilation, resulting in superior radiation tolerance for the material to be used in applications such as advanced fusion reactors.
